# Identification and analysis of a novel A allele with a c.565A > G variant on the ABO*A1.02 allele leading to subtype A: A case report

**DOI:** 10.1097/MD.0000000000046872

**Published:** 2025-12-26

**Authors:** Weiwei Lu, Chunping Mo, Jing Zhong, Yan Zhang, Lingbo Li

**Affiliations:** a Department of Blood Transfusion, The First Hospital of Jilin University, Changchun, China; b Department of Blood Transfusion, The Central Hospital of Shaoyang, Shaoyang, China; c Research and Development Department, Changchun Bioxun Biotechnology Limited Liability Company, Changchun, China; d Research and Development Department, Tianjin Dexiang Biotechnology Co., Ltd, Tianjin, China.

**Keywords:** ABO blood group, genotype, PCR, sequencing, subtype A, TA cloning

## Abstract

**Rationale:**

The ABO blood group system typically includes 4 phenotypes: type A, type B, type O, and type AB. ABO subtypes refer to further subdivisions within these 4 blood types. We report a case of subtype A, focusing on elucidating the molecular mechanisms of this rare genotype.

**Patient concerns:**

The patient is a 34-year-old pregnant woman at 39 weeks gestation presenting with preterm labor. She exhibits forward and reversed stereotypic incompatibility.

**Diagnoses:**

All exons of ABO were amplified by PCR and sequenced using Sanger sequencing. Furthermore, only the seventh exon was sequenced by TA cloning for haplotype analysis. Sequencing analysis revealed that the genotypes ABO*A1.02 and ABO*O.01.02, and the mutation c.565A > G, which has never been reported for the A allele, occurred based on ABO*A1.02.

**Interventions:**

In addition to serological test, the patient underwent further molecular biology test.

**Outcomes:**

It led to identify a novel allele with subtype A serological characteristics, caused by the c.565A > G variant, which leads to a weakened A antigen.

**Lessons:**

Given its rarity, it is advisable to employ dual serological and molecular biological testing mechanisms during complex ABO blood typing to prevent missed detection and misclassifications, thereby effectively promoting transfusion and compatibility safety.

## 1. Introduction

The ABO blood group system is the first of the human blood groups to be discovered and is still the most important blood group system in clinical practice.^[1–3]^ ABO blood group identification is of significant importance in clinical blood transfusion and organ transplantation.^[4,5]^ Accurate matching is the primary prerequisite to ensure the safe and effective utilization of blood, tissue, and organ resources, so ABO blood group genetic identification is gradually becoming the new standard. The need for the identification of complex blood groups in the clinic, especially subtypes and novel allelotypes never reported before, can be well addressed using molecular biology techniques. In the present study, a novel A allele with a c.565A > G variant leading to subtype A was identified during genetic identification of complex blood groups using PCR amplification, Sanger sequencing, and TA cloning techniques.

## 2. Case presentation

### 2.1. Materials and methods

#### 2.1.1. Objects

The proband was a 34-year-old pregnant woman at 39 weeks gestation with forward and reversed stereotypic incompatibility undergoing preoperative blood preparation at the Central Hospital of Shaoyang with no previous history of blood transfusion. She has experienced vaginal bleeding for over 12 hours, abdominal pain for over 6 hours, indicating preterm labor. This study followed the basic principles of medical ethics and was approved by the hospital’s Medical Ethics Committee (No. KY 2023-002-25), and informed consent was waived.

#### 2.1.2. Instruments and reagents

ABO and RhD blood group forward typing test card (monoclonal antibody), ABO blood group reversed typing kit (human red blood cell), both produced by Changchun Bioxun Biotech Co., Ltd; UE Blood Genomic DNA Small Volume Preparation Kit, produced by Suzhou UElandy Biotechnology Co.; Primers for exons 1 to 7 of the *ABO* gene, produced by Comate Bioscience Co., Ltd.; TD-A type centrifuge for blood group serology, produced by Changchun Bioyan Scientific Instrument Co., Ltd; Centrifuge 5425 micro centrifuge and Nexus GX2 PCR instrument, both produced by Eppendorf Life Sciences; And 3730XL gene sequencer, produced by Thermo Fisher Scientific. All reagents were used within the expiration date and all instruments were used after calibration.

#### 2.1.3. Methods

##### 2.1.3.1. Serological identification method

A test card was used for identification and strictly followed the manufacturer’s instructions. The absorption-elution test was carried out in strict accordance with the AABB Technical Manual.^[6]^

##### 2.1.3.2. Molecular biological identification method

PCR amplification, Sanger sequencing, and TA cloning techniques were used to identify the genotype of the proband with a complex blood group. Genomic extraction: DNA was extracted from the proband’s EDTA-anticoagulated whole blood using the kit following the manufacturer’s instructions. PCR amplification: Based on the sequence information NC_000009.12 and NM_020469.3 provided by the National Center for Biotechnology Information and the reference,^[7,8]^ we designed 7 pairs of amplification primers for exons 1 to 7 of the *ABO* gene, and the amplification primers were consistent with the sequencing primers. The PCR reaction system was as follows: 50µL dNTP-Buffer Mixture (Mg^2+^ plus) with 0.5 µL Taq (5 U/µL), followed by 2µL of amplification primer (10 µM) and 2 µL of genomic DNA (100 ng/µL). The PCR amplification program was as follows: 94°C for 5 minutes, 1cycle; 94°C for 30s, 55°C for 30s, 72°C for 60s, 35cycles; 72°C for 5 minutes, 1cycle. PCR product identification: The above 7 products were detected using 1% agarose gel electrophoresis . Sequencing: The 7 products were sequenced using Sanger sequencing, and TA clone sequencing was added to the exon 7 amplification product. Genetic analysis: According to the “Names for ABO (ISBT 001) blood group alleles v1.1 171023” blood group allele table published by the International Society of Blood Transfusion (ISBT), using ABO*A1.01 as the reference sequence, the sequencing results were analyzed with the bioinformatics software SnapGene and EditSeq to determine the ABO blood group genotype.

## 3. Results

### 3.1. Serological test results

**Figure 1.**
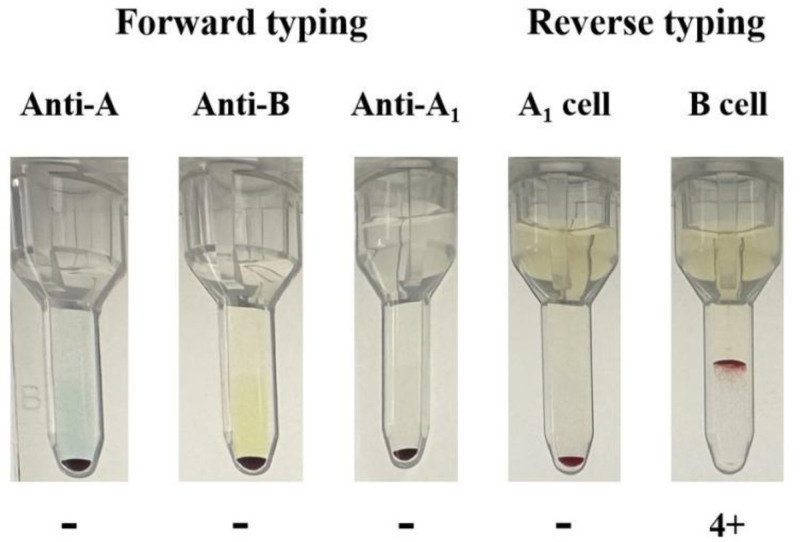
Serological test results: “−” indicates no agglutination, “+” indicates agglutination (“4+” indicates strong agglutination).

The proband’s red blood cells reacted with anti-A, anti-B and anti-A_1_ without agglutination, and the forward stereotype was “O.” The proband’s plasma reacted with A_1_ reagent red blood cells without agglutination and no anti-A_1_ antibody was detected, while it reacted with B reagent red blood cells with 4 + agglutination, and the reversed stereotype was “A.” The absorption-elution test detected a weak A antigen on the proband’s red blood cells, and the serotype was initially determined to be subtype A (Fig. 1).

### 3.2. Molecular biology test results

**Figure 2.**
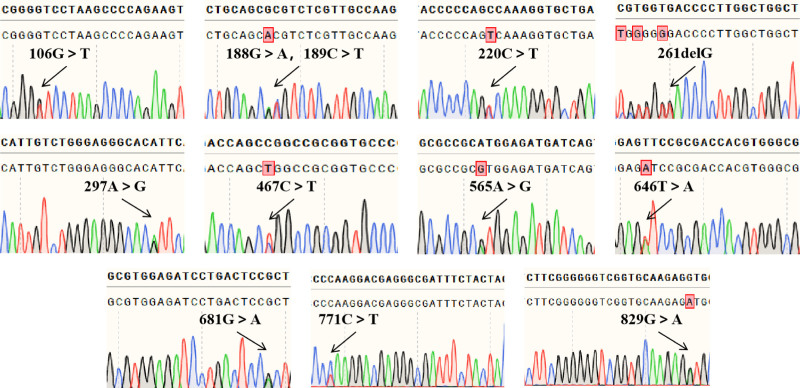
Sanger sequencing results: it can be seen that 12 mutations in exons 3 through 7 (heterozygous mutation).

**Figure 3.**
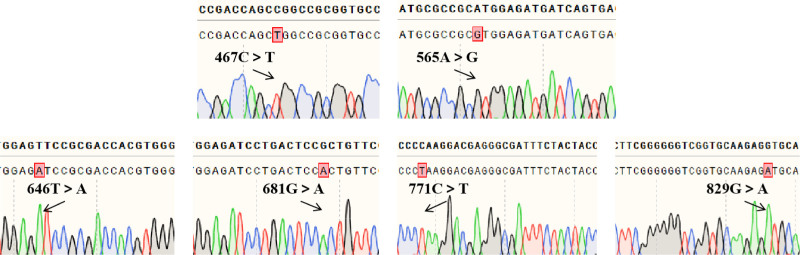
TA clone sequencing results: it can be seen that 6 mutations in exon 7 (homozygous mutation).

The 7 PCR amplification products of exons 1 to 7 of the *ABO* gene, electrophoresis showed unique bands that were clearly recognizable and the size of the product fragments matched the design, indicating that the target fragments were amplified. Sanger sequencing showed no mutations in exons 1 and 2, and mutations in exons 3 through 7, with the following nucleotide changes: c.106G > T, c.188G > A, c.189C > T, c.220C > T, c.261delG, c.297A > G, c.467C > T, c.565A > G, c.646T > A, c.681G > A, c.771C > T, and c.829G > A (Fig. 2). Furthermore, TA clone sequencing of exon 7 showed that c.467C > T and c.565A > G occurred on the same strand, and c.646T > A, c.681G > A, c.771C > T, and c.829G > A occurred on the same strand (Fig. 3). The combined analysis determined the genotypes to be ABO*A1.02 and ABO*O.01.02 with the c.565A > G variant on ABO*A1.02, which resulted in a weakened A antigen and manifested subtype A serological characteristics.

## 4. Discussion

The ABO blood group system was first discovered in 1900, and the identification of genotypes was revealed through studies at the molecular level.^[9]^ That is, the *ABO* gene and its 7 coding exons give rise to 2 principally different glycosyltransferases, the A glycosyltransferase (GTA) and the B glycosyltransferase (GTB). As a result, genetic polymorphisms give rise to 2 related antigens in this system, and any polymorphism that changes the activity of the enzyme may alter the ABO phenotype. Alterations that completely abolish the activity of the enzyme give rise to the O phenotype, in which the H antigen remains unconverted and no A or B antigens can be detected. If alterations decrease the activity of the enzyme, and thus decrease the conversion of H to A or B, a weak A or B phenotype can result. The most common subtypes of blood type A are mainly A_1_ and A_2_, which account for more than 90% of all A blood groups, while the uncommon subtypes A_3_, A_weak_, A_x_, A_m_ and A_el_, have progressively weaker antigenic expression. The typical serological characteristics of subtype A are that red blood cells do not agglutinate or agglutinate weakly with the anti-A antibody. The same was true for patients in this study, where serological tests revealed a discrepancy between forward and reversed stereotypes, which were analyzed by an absorption-elution test and initially determined to be subtype A. Therefore, serology cannot accurately type subtypes, and molecular biology is needed to solve the problem of identifying difficult blood types.

Compared with normal blood groups, subtypes differ in terms of antigenic structure, properties, and quantity. The patient in this study was genotyped for the ABO blood group and was found to have mutations in exons 3 to 7, mainly in exons 6 and 7. The genotypes were ABO*A1.02 and ABO*O.01.02, with the c.565A > G variant occurring based on ABO*A1.02. According to the ABO blood group allele table by ISBT, no subtype A formed as a result of the c.565A > G mutation was identified (https://www.isbtweb.org/resource/001aboalleles.html). Analysis suggested that the c.565A > G mutation changes position 189 of the encoded transferase from methionine (Met) to valine (Val),^[10]^ altering glycosyltransferase activity and resulting in a weakened A antigen. This elucidates the molecular mechanism leading to antigenic attenuation, while the absorption-elution test results validate the serological characteristics.

The main causes of complex blood groups in the clinic are pathologic interference, physiologic interference, transfusion interference, and subtypes, all leading to forward and reversed stereotypic incompatibility and affecting the result of transfusion compatibility testing. First, it is important to ensure that clinical laboratory personnel operate in a standardized manner, and if necessary, repeat the operation to verify, and then consider the presence of interference factors. Second, clinical laboratory personnel should be equipped with analytical methods for solving such problems. The process of identifying the patient’s blood group in this study was similar; the clinical laboratory personnel performed the test in strict accordance with the norm, ruling out the presence of interference in the patient itself first, and then reasonably suspecting the subtype later. After the initial serological determination of subtype A, molecular biology identified a novel A allele with c.467C > T and c.565A > G.

In actual clinical practice, subtypes are often the main cause of difficulties in serological identification or even misclassification, and therefore are highly susceptible to hemolytic reactions.^[11–14]^ ABO subtypes, as more specific phenotypes, have a very low detection rate in the Chinese population, and the detection rate of subtype A is extremely low.^[15,16]^ At the present time, blood group serological identification is routine, but sometimes, it may lead to missed detection or misclassification.^[17]^ Molecular biology identification can eliminate the interference of complex blood groups, make up for the limitations of serological identification, and allow precise determination of subtypes.^[16]^ The results of the patient’s molecular biological identification in this study have important clinical implications, revealing a novel allele with subtype A serological characteristics caused by the c.565A > G variant.

The precise determination of patients’ ABO blood group identification can provide a strong scientific basis for clinical precision blood transfusion, which can help clinical blood transfusion and organ transplantation, improve the safety of blood transfusion, and promote the development of precision medicine.^[11,18]^ In this study, the patient was only tested for exons 1 to 7 of the *ABO* gene by Sanger sequencing, which has some limitations. It has been shown that third-generation sequencing analysis reveals the presence of novel intron mutations in subtype A_el_ (c.467C > T; c.29-10T > A) and novel transcription factor binding site in subtype A_3_ (+5.8 kb site).^[19,20]^ It can be seen that Sanger sequencing is the basis for solving difficult blood group identification and third-generation sequencing is the trend.

In summary, this study suggests the need for a dual test mechanism that can be used in difficult ABO blood group identification through serological identification and molecular biological identification to avoid missed detection and misclassification and to effectively promote blood transfusion and matching safety.

## Acknowledgments

This work was supported by the Health Research Project of Hunan Provincial Health Commission (grant number: 20253937), the Shaoyang Science and Technology Plan Project (grant number: 2023ZD0106), and the Scientific Research Project of Hunan Provincial Health Commission (grant number: 202211003190).

## Author contributions

**Data curation:** Weiwei Lu.

**Formal analysis:** Chunping Mo.

**Funding acquisition:** Jing Zhong.

**Project administration:** Weiwei Lu, Chunping Mo.

**Supervision:** Jing Zhong, Yan Zhang, Lingbo Li.

**Writing – original draft:** Yan Zhang.

**Writing – review & editing:** Jing Zhong, Yan Zhang, Lingbo Li.
